# Chromosome number and ploidy level of balm (*Melissa officinalis*)

**DOI:** 10.1186/s13039-015-0166-z

**Published:** 2015-08-07

**Authors:** J. Kittler, O. Schrader, U. Kästner, F. Marthe

**Affiliations:** Institute for Breeding Research on Horticultural Crops of the Julius Kuehn Institute (JKI), Federal Research Centre for Cultivated Plants, Erwin-Baur-Str. 27, Quedlinburg, D-06484 Germany

## Abstract

**Background:**

Lemon balm (*Melissa officinalis* L.) is of increasing importance resulting in rising growth area. Improved knowledge on the genome structure, number of chromosomes in connection with the taxonomical structure of balm is indispensable for improved new varieties.

**Results:**

A collection of 40 balm accessions (*M. officinalis*) was characterized by flow cytometry and FISH (18/25S and 5S rDNA) to determine the chromosome number and ploidy level. Three different types were found: diploid genotypes with 2n = 2× = 32 chromosomes; tetraploid 2n = 4× = 64 chromosomes and triploid 2n = 3× = 48 chromosomes. A haploid base number of × = 16 chromosomes is likely. First time described triploid accessions are sterile but cytologically and morphologically stable for many years. Triploids express better winter hardiness and regeneration after harvesting cuts as well as bigger leaves and internodes.

**Conclusions:**

A basic chromosome number of x = 16 is reported for the first time for the species *M. officinalis*.

## Abstract (de)

Die wachsende Bedeutung von Zitronenmelisse (*Melissa officinalis* L.) führt zur Ausdehnung des hierfür erforderlichen Anbauumfanges. Ein verbesserter Kenntnisstand der Genomstruktur, der Chromosomenzahl und der hiermit in Zusammenhang stehenden taxonomischen Struktur der Melisse sind unerlässliche Voraussetzungen für verbesserte, neue Sorten.

Eine Kollektion von 40 Melisseherkünften (*M. officinalis*) wurde durchflusszytometrisch und durch FISH (18/25S and 5S rDNA) untersucht, um den Ploidiegrad und die Chromosomenzahl zu ermitteln. Drei unterschiedliche Typen wurden konnten bestimmt werden: diploide Genotypen mit 2n = 2× = 32 Chromosomen; tetraploide mit 2n = 4× = 64 Chromosomen und triploide mit 2n = 3× = 48 Chromosomen. Die haploide Chromosomenzahl ist mit x = 16 anzunehmen. Die erstmalig beschriebenen triploiden Herkünfte sind steril aber zytologisch und morphologisch über viele Jahre stabil. Sie zeigen eine bessere Winterhärte und einen schnelleren Wiederaufwuchs nach Ernteschnitten, wie auch größere Blätter und Internodien.

Die Basischromosomenzahl von x = 16 wird erstmalig für die Art *M. officinalis* beschrieben.

## Background

Lemon balm (*Melissa officinalis* L.) is an old crop and is used for phytopharmaceuticals, as an aromatic plant and in traditional folk medicine. A wide spectrum of secondary metabolites exists in lemon balm. For the medicinal use, the active ingredients essential oil with lemon fragrance and rosmarinic acid are necessary [6, 2, 3, 13].

*M. officinalis* belongs to the family Labiatae (syn. Lamiaceae). This crop plant is grown worldwide but its origin is not well-defined, however the Mediterranean Region or western Asia is considered as the area of origin [9]. The subspecies *M. officinalis* L. ssp. *officinalis* and ssp. *altissima* (Sibth. & Sm.) Arcangeli are distinguishable especially by the shape of calyx and density of different types of hairs [5, 20]. The middle tooth of the three upper lip teeth of fruiting calyx is broadly triangular for ssp. *officinalis* whereas it is inconspicuous, truncate or emarginated for ssp. *altissima*. Pignatti [17] classified ssp. *altissima* as species *M. romana*.

The chromosome base number in Labiatae ranges from x = 5 to 11, but also x = 13, 15, 17 and 19 occur [10]. For higher numbers, polyploidy is assumed followed by structural rearrangements. Chromosome numbers of 32 and 64 were reported for *M. officinalis* ssp. *officinalis* and ssp. *altissima*, respectively [20]. Ssp. *altissima* has been suggested as an ancestor of the cultivated diploid ssp. *officinalis* despite being tetraploid [9]. Davis [5] separated the ssp. *inodora* (Bornm.) Bornm. based on the long patent hairs on stems and fairly distinct, triangular middle tooth of upper calyx lip and mentioned intermediates between all three subspecies. Pignatti [17] described beside *M. officinalis* L. (2n = 32) the species *M. romana* Miller as synonymous with ssp. *altissima* because of the absence of any offspring (2n = 64). Darlington and Wylie [4] presented the haploid chromosome number for *M. officinalis* with x = 8.

Lemon balm is of increasing importance, resulting in rising growth area and investigations for improved new varieties. Better winter hardiness, higher content of essential oil and higher yield of *M. folium* (lemon balm leaves) are of special interest in these programmes. Adaptation of technologies for acceleration of the breeding process using doubled haploid lines has been initiated. Therefore, the knowledge on the genome structure and number of chromosomes in connection with the taxonomical structure of *M. officinalis* is indispensable. In order to determine the ploidy level of 40 accessions and the chromosome number of the haploid *M. officinalis* genome, we employed flow cytometry and FISH using rDNA-specific probes. Beside di- and tetraploid accessions, first time triploid accessions (2n = 3× = 48) have been identified for *M. officinalis*.

## Results and discussion

Genome size determination by flow cytometry of *M. officinalis* revealed for 23 accessions a diploid, four accessions a triploid and 13 accessions a tetraploid ploidy level (Table 1).

**Table 1:** Determined ploidy level of balm accessions (*Melissa officinalis*) from Leibniz institute for plant genetics and crop plant research at Gatersleben, Germany (IPK) and bavarian state institute for agriculture at Freising, Germany (LfL) based on flow cytometry

Accession No.	COL^a^	Taxonomical group of *Melissa officinalis* L.^b^	Origin^b^	Ploidy level
BLBP 5	LfL	*M. officinalis* L.	southern France	diploid
BLBP 8	LfL	*M. officinalis* L.	Spain	diploid
BLBP 19	LfL	*M. officinalis* L.	Germany	diploid
BLBP 27	LfL	*M. officinalis* L.	Germany	diploid
BLBP 33	LfL	*M. officinalis* L.	botanical garden Halle, Germany	diploid
BLBP 48	LfL	*M. officinalis* L.	Germany	diploid
BLBP 49	LfL	*M. officinalis* L.	Unknown	diploid
BLBP 50	LfL	*M. officinalis* L.	Unknown	diploid
BLBP 78	LfL	*M. officinalis* L.	France	triploid
BLBP 87	LfL	*M. officinalis* L.	Georgia, landrace	diploid
BLBP 88	LfL	*M. officinalis* L.	botanical garden Liege, France	triploid
BLBP 111	LfL	*M. officinalis* L.	Hungary	triploid
BLBP 113	LfL	*M. officinalis* L.	France	triploid
MELI 1	IPK	*M. officinalis* L.	Unknown	diploid
MELI 2	IPK	*M. officinalis* L.	Unknown	diploid
MELI 4	IPK	*M. officinalis* L.	East Germany	diploid
MELI 5	IPK	*M. officinalis* L. ‘Erfurter Aufrechte’	East Germany	diploid
MELI 6	IPK	*M. officinalis* L.	Germany	diploid
MELI 7	IPK	*M. officinalis* L. ‘Citra’	Unknown	tetraploid
MELI 8	IPK	*M. officinalis* L.	Georgia	diploid
MELI 10	IPK	*M. officinalis* L.	France	diploid
MELI 11	IPK	*M. officinalis* L. ‘ital. Melissa, Cedronella’	Italy	diploid
MELI 12	IPK	*M. officinalis* L.	Italy	tetraploid
MELI 13	IPK	*M. officinalis* L.	Georgia	diploid
MELI 14	IPK	*M. officinalis* L. subsp. *altissima* (Sibth. & Sm.) Arcang.	Italy	tetraploid
MELI 15	IPK	*M. officinalis* L. subsp. *altissima* (Sibth. & Sm.) Arcang.	Italy	tetraploid
MELI 16	IPK	*M. officinalis* L. subsp. *officinalis*	Unknown	diploid
MELI 17	IPK	*M. officinalis* L.	Greece	tetraploid
MELI 18	IPK	*M. officinalis* L. subsp. *altissima* (Sibith. & Sm.) Arcang.	Unknown	tetraploid
MELI 19	IPK	*M. officinalis* L.	Italy	tetraploid
MELI 20	IPK	*M. officinalis* L.	Italy	tetraploid
MELI 21	IPK	*M. officinalis* L. subsp. *altissima* (Sibth. & Sm.) Arcang.	Albania	tetraploid
MELI 22	IPK	*M. officinalis* L.	Turkey	tetraploid
MELI 23	IPK	*M. officinalis* L. subsp. *altissima* (Sibth. & Sm.) Arcang.	Italy	tetraploid
MELI 24	IPK	*M. officinalis* L. subsp. *altissima* (Sibth. & Sm.) Arcang.	Italy	tetraploid
MELI 25	IPK	*M. officinalis* L. ‘Zitronenmelisse’	Unknown	diploid
MELI 26	IPK	*M. officinalis* L.	Armenia	diploid
MELI 27	IPK	*M.* sp.	Italy	diploid
MELI 28	IPK	*M. officinalis* L.	Italy	tetraploid
D9597	IPK	*M.* sp. ‘Zitronenmelisse’	Unknown	diploid

^a^COL: collection

^b^taxonomical classification according to information of collection holders

**Fig. 1:**
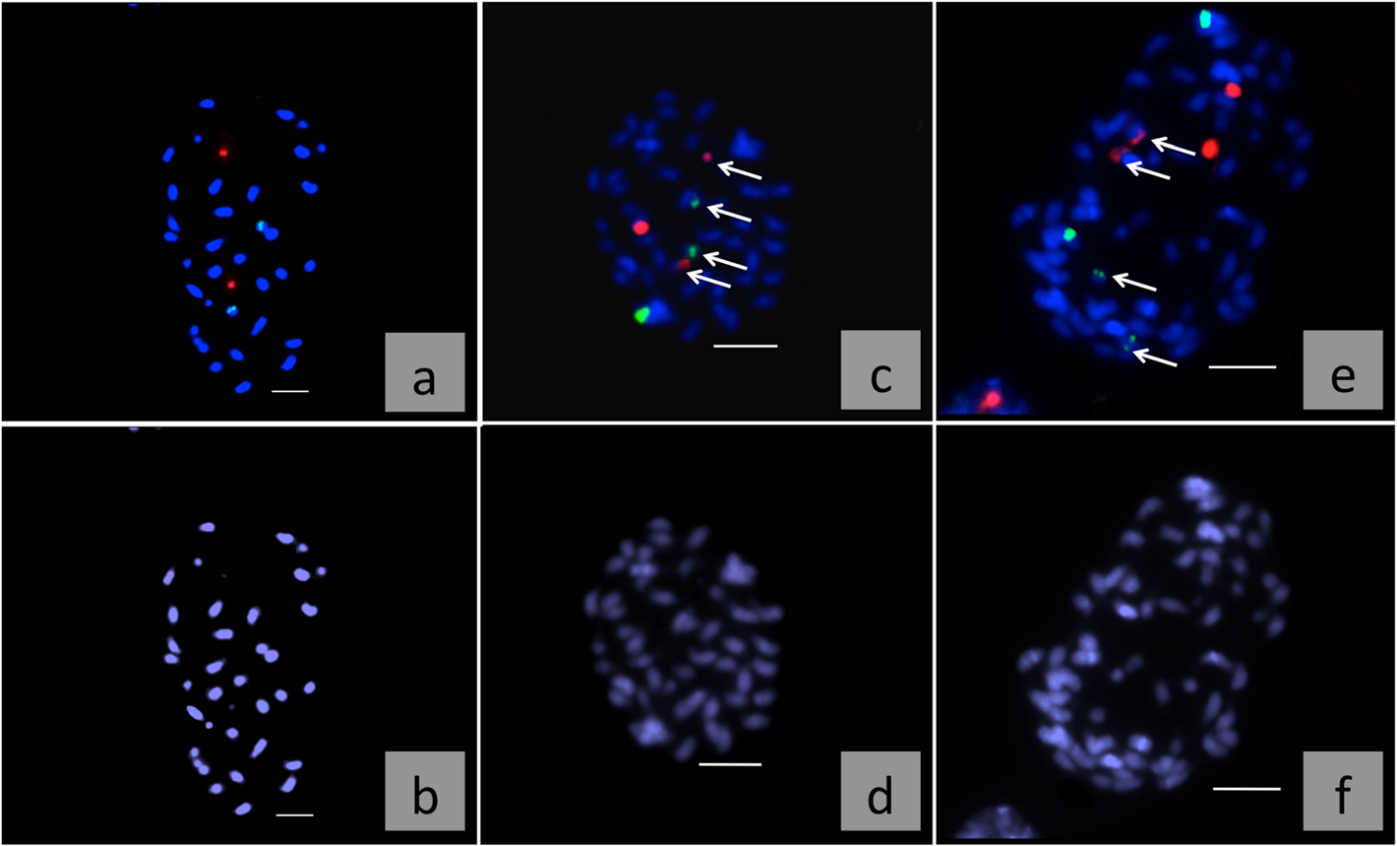
**a-f**: Mitotic metaphase chromosomes of balm (*Melissa officinalis*) after FISH with 18/25S rRNA and 5S rRNA-specific probes. Above (**a**, **c**, **e**): FISH, red 18/25S rDNA, green 5S rDNA, arrows mark weak signals, below (**b**, **d**, **f**): DAPI stained chromosomes; a: diploid MELI 1, 2n = 2x = 32; c: triploid BLBP 78, 2n = 3x = 48, e: tetraploid MELI 22, 2n = 4x = 64. The size bars equals 5 μm

To confirm the ploidy predictions, chromosome numbers were determined and multicolour FISH was performed with 18/25S- and 5S rDNA-specific probes (Table 2). In *M. officinalis* 5S and 18/25S rDNA were localized on different chromosomes. Unlike this result in some other genera e.g. *Helianthus, Brassica* and *Alstroemeria* [18, 19, 1] localization of 5S and 18/25S rDNA on the same chromosome was found. Analysis of six selected accessions revealed for the putative diploid genotypes a chromosome number of 32 and two chromosome pairs exhibiting either 18/25S rDNA- or 5S rDNA-specific signals (Fig. 1a, b). Putative triploid accessions contained 48 chromosomes and revealed six distinct hybridization signals: three 18/25S rDNA and three 5S rDNA (Fig. 1c, d). The last category exhibited 64 chromosomes and eight signals: four 18/25S rDNA and four 5S rDNA sites (Fig. 1e, f).

**Table 2:** Level of ploidy, number of chromosomes and signals resulting from FISH with 18/25S rDNA and 5S rDNA probes in accessions of balm (*Melissa officinalis*)

Accession	Number of chromosomes	Signals of 18/25S rDNA	Signals of 5S rDNA	Ploidy
BLBP 48	32	2	2	diploid
MELI 1	32	2	2	diploid
BLBP 78	48	3	3	triploid
BLBP 113	48	3	3	triploid
MELI 14	64	4	4	tetraploid
MELI 22	64	4	4	tetraploid

In accessions showing six or eight hybridization signals, the intensity of rDNA signals varied. Metaphases of accessions showing six signals displayed one strong and two weak 18/25S rDNA signals (Fig. 1c). The same was true for 5S rRNA sites. Metaphases of genotypes showing eight rDNA signals for each marker, one chromosome pair displayed a strong and one pair a weak hybridization signal (Fig. 1e). Hence accessions with 32 chromosomes and one pair of 18/25S rDNA and 5S rDNA signals are diploid (2n = 2× = 32). Accessions with 48 chromosomes and three rDNA signals are triploid (2n = 3× = 48), and accessions with 64 chromosomes and two pair of rDNA signals are tetraploid (2n = 4× = 48). Therefore, a chromosome basic number of x = 16 in the genus *Melissa* is likely. In contrast, Darlington and Wylie [4] postulated a basic chromosome number of x = 8 and a somatic number of 2n = 32, without giving any information about investigated subspecies. Later on, Tutin et al. and Pignatti [20, 17] reported a chromosome number of 2n = 32 for *M. officinalis* ssp. *officinalis. M. officinalis* ssp. *altissima* and *M. romana* has 2n = 64 chromosomes [17, 20]. The reports of Heidari et al. [11] and Murin [15] of 2n = 32 chromosomes and Löve [14] of 2n = 64 chromosomes for *M. officinalis* provide no information about the analysed subspecies.

Two scenarios regarding the origin of triploid balm can be postulated: an unreduced gamete of a diploid plant formed triploid offspring after fertilization with a haploid gamete. Alternatively, a tetraploid parent hybridized with diploid parent and formed triploid offspring. The different signal intensity of either 5S or 18/25S rDNA sites in triploid balm could be explained by the different copy number of parental rDNA repeats.

### Phenotype characterization of triploid balm

**Fig. 2:**
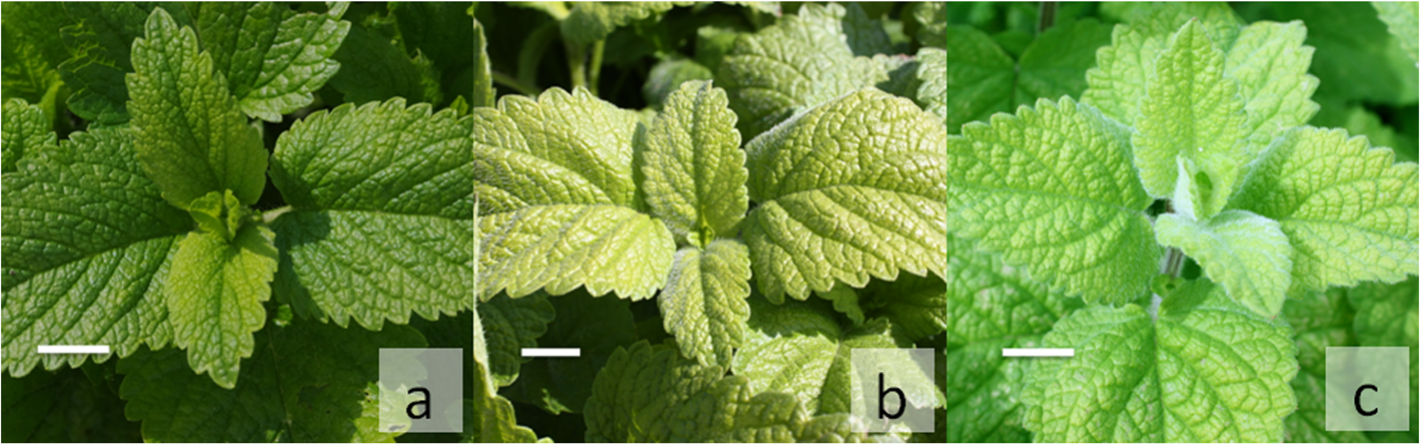
leaf colour and density of pubescence in balm (*Melissa officinalis*); **a**: diploid, **b**: triploid and **c**: tetraploid. The size bars equals 1 cm

The plant phenotype of the four triploid accessions was characterized. Stems of the specimen BLBP 78, BLBP 88, BLBP 111 and BLBP 113 reached 120 to 140 cm (ssp. *officinalis* 50 to 80 cm, [17]) with tendency of laying down and entangling. The size of leaves (7.6 cm length, standard deviation s 0.59 and 5.4 cm width, s 0.56) was bigger than diploid ssp. *officinalis* type leaves (6.9 cm length, standard deviation s 0.51 and 4.5 cm width, s 0.42). The internodes are longer (9.4 cm, standard deviation s 0.99) in comparison with diploid ssp. *officinalis* (6.5 cm, s 0.78) accessions (Table 3). Triploid accessions had very good cold resistance and regenerated faster after winter and harvesting cuts (results not shown). The colour of leaves was bluish to greyish green in comparison to green leaves of ssp. *officinalis* (Fig. 2). Young leaves of triploid accessions have indumenta similar to ssp. *altissima* (Fig. 2) whereas according to Tutin et al. [20], leaves of diploid ssp. *officinalis* are glabrescent or sparsely hairy above, glandular-puberulent and more or less sparsely hairy beneath. Adult leaves of triploid accessions are more similar to ssp. *officinalis*. Stems of triploid accessions are greyish- or whitish-tomentose beneath with similarity to ssp. *altissima*. The triploid accessions are not lemon-scented. They had a soap-like, nauseating scent. These plants had an inconspicuous formation of flowers but do not produce any seed, neither under conditions of isolation nor by open pollination, likely due to meiotic problems. These first time described triploid accessions were propagated by cuttings and are cytologically and morphologically stable for at least six years.

**Table 3:** Length and width of second leaf and length of second internode from base of stem for balm (*Melissa officinalis*) of different ploidy level

ploidy	leaf length [cm]				leaf width [cm]				internodes [cm]				n^b^
level	Min	mean	max	s^a^	min	mean	max	s^a^	min	mean	max	s^a^	
diploid	6.2	6.9	7.5	0.51	4.0	4.5	5.1	0.42	5.8	6.5	8.3	0.78	9
triploid	6.9	7.6	8.1	0.59	4.7	5.4	6.0	0.56	8.4	9.4	10.5	0.99	4

s^a^ standard deviation

n^b^ number of accessions; For each accession at least ten plants were measured

## Conclusions

The basic chromosome number of x = 16 is reported for the first time for the species *M. officinalis* and for family Labiatae.

This is the first characterization of triploids in *M. officinalis*. These triploid accessions are sterile but cytologically and morphologically stable. The length and width of the leaves and the length of internodes exceeded the comparable data for diploid accessions but are not significant for all characters.

For exact origin analysis of triploids as well as the characterization of allotetraploid or autotetraploid character of tetraploids analysis of meiotic chromosome pairing is necessary. Chromosome specific molecular markers would open the chance to ascertain the level of similarity of homoeologous groups of chromosomes. This is a prerequisite for better characterization of phylogenetic distance of *M. officinalis* ssp. *altissima* in comparison to ssp. *officinalis*.

## Material and methods

### Plant materials

A set of 40 accessions of *M. officinalis* have been characterized, of which 27 and 13 were provided from the Federal *ex-situ* Collection of Agricultural and Horticultural Plants of the Leibniz Institute for Plant Genetics and Crop Plant Research at Gatersleben, Germany (IPK) and the Bavarian State Institute for Agriculture at Freising, Germany (LfL) respectively. LfL collection contained old varieties and breeding material from middle and western Europe, the IPK collection includes mainly landraces and wild types from eastern and middle Europe (Table 1). All IPK accessions were grown from seeds whereas all LfL accessions were propagated by cuttings starting with a single plant. Radish (*Raphanus sativus* L.) was used as genome size marker in flow cytometry.

### Evaluation of ploidy level by flow cytometry

Measuring of relative DNA amount of nuclei occurred by flow cytometry (Facs calibur, Becton Dickinson, BD) with a red fluorescence laser as basis for detection of ploidy level. For each probe, leaf material was chopped with razor blades in 500 μl nuclei extraction buffer (CyStain PI absolute P, Sysmex) and stained with the corresponding staining buffer, containing 5 % polyvinylpyrrolidone 25 (Serva) and 0.6 % propidium iodide (Serva). Immediately after staining, the nuclei suspension was filtered using a 5 ml polystyrene round-button tube with a cell-strainer cap (BD). For reference, radish was measured in separate sample after five samples of balm.

### Chromosome preparation

*M. officinalis* seeds were germinated and the cell division synchronized with 1.25 mM hydroxyurea for 17 h. For vegetative multiplied accessions (LfL), root tips from potted plants were used. In contrast to Pan et al. [16], the recovery time after hydroxyurea treatment was 24 h at 6 °C. Root tips were fixed in ethanol-acetic acid (3:1, 24 h) and stored in 70 % ethanol at −20 °C. After washing with aqua dest. root tips were digested with an enzyme mixture (4 % cellulase, ‘Onozuka R-10’, Serva and 1 % pectlyase Y-23 (Seishin Pharmaceutical)) in 75 mM KCl and 7.5 mM Na-EDTA, (pH 4.0 for 36 min. at 37 °C, [12]). Softened root tips were squashed in 45 % acetic acid. After removal of the coverslip by freezing (−84 °C) the slides were air dried overnight at 24 °C and stored at −20 °C.

### Fluorescence *in situ* hybridization

The 18S-5.8S-25S rDNA loci were detected with a 220 bp-long 25S repeat-specific probe labelled with biotin-16-dUTP (Boehringer Mannheim) during polymerase chain reaction (PCR) amplification of the genomic DNA of *Allium ampeloprasum* with primers designed according to the sequence published by Yokota et al. [21]. For the localization of 5S rRNA genes, a 117 bp fragment obtained after PCR amplification from the same genomic DNA with specific primers coding for this region [8] was used. The labelling of this amplified probe was performed with digoxigenin-11-dUTP (Boehringer Mannheim). The hybridization mixture contained 80 ng of each DNA probe (5S and 25S rDNA) and 10 μg of salmon-sperm DNA in 20 μl of hybridization buffer (50 % deioinized formamide, 10 % dextran sulphate, 2 x SSC) per slide [19].

The FISH procedure was performed according to Fuchs and Schubert [7] with the following modifications: prior to hybridization, slides were incubated in 50 ng/μl of DNase-free RNase in 2 x SSC for 1 h at 37 °C, washed three times in 2 x SSC for 5 min and treated with 0.5 ng/μl of proteinase K for 10 min at 37 °C, followed by three times washing in 2 x SSC for 15 min. The slides were then postfixed in 4 % paraformaldehyde for 10 min, washed three times in 2 x SSC for 15 min, dehydrated in a graded ethanol series (70, 80 and 96 %) at −20 °C, and air-dried. The hybridization mixture (probe) was denaturated (80 °C, 7 min), incubated on ice (about 5 min), dropped onto slides, covered with coverslips, and sealed with rubber cement. Probes and chromosomes were denaturated together on a heated desk (7 min, 80 °C). The slides were then incubated overnight at 37 °C in a humidity chamber. After hybridization and removing the coverslips, the slides were washed in 2 x SSC at 37 °C three times for 5 min each, followed by three 5 min stringent washes in 0.3 x SSC at 60 °C and then blocked for 30 min at 37 °C with a solution of 4 x SSC, 3 % BSA and 0.1 % Tween 20. The biotinylated probe was detected with 10 ng/μl of streptavidin-Cy3 (Dianova) and amplified with two steps of 10 ng/μl of biotinylated anti-streptavidin (Vector) and 10 ng/μl strepavidin-Cy3. Together with the first amplification step of the biotin labelled probe, the detection of the digoxigenin labelled probe with 9 ng/μl of anti-digoxigenin-fluorescein (Roche) was done and then amplified with 8 ng/μl anti-sheep-fluorescein (Dianova). Chromosomes were counterstained and embedded in 15 μl of DAPI-VECTASHIELD antifade solution (Vector Laboratories). Images were captured for each fluorescence dye separately with a cooled CCD camera system Axiocam (Zeiss) on a microscope Axioimager Z1 (Zeiss) with the following filter combinations: 02 (DAPI), 10 (FITC) and 20 (Cy3). Pseudocoloration and mergence of images were done with software of the Isis program (Metasystems).
